# 

**Published:** 1921-12

**Authors:** 


*Vol. XXXVIII.* *No.* 144.

THE

### BRISTOL MEDICO-CHIRURGICAL JOURNAL

*PUBLISHED UNDER THE AUSPICES OF*

*THE BRISTOL MEDICO-CHIRURGICAL SOCIETY.*

*Editor:* P. WATSON-WILLIAMS, M.D.,

WITH WHOM ARE ASSOCIATED

JAMES SWAIN, C.B., C.B.E., M.S., M.D.,

J. LACY FIRTH, M.S., M.D., CAREY COOMBS, M.D.

J. A. NIXON, C.M.G., M.D., *Assistant Editor.*

*Editorial Secretary:* A. L. FLEMMING, M.B., Ch.B.

BRISTOL: J. W. ARROWSMITH LTD.

LONDON: SIMPKIN, MARSHALL, HAMILTON, KENT & Co. LTD.

*Price Three Shillings net.*

